# Broca's area and its striatal and thalamic connections: a diffusion-MRI tractography study

**DOI:** 10.3389/fnana.2013.00008

**Published:** 2013-05-10

**Authors:** Anastasia A. Ford, William Triplett, Atchar Sudhyadhom, Joseph Gullett, Keith McGregor, David B. FitzGerald, Thomas Mareci, Keith White, Bruce Crosson

**Affiliations:** ^1^Department of Veterans Affairs Rehabilitation Research and Development Brain Rehabilitation Research Center, Malcom Randall VA Medical CenterGainesville, FL, USA; ^2^Department of Psychology, University of FloridaGainesville, FL, USA; ^3^Department of Biochemistry and Molecular Biology, University of FloridaGainesville, FL, USA; ^4^Department of Neurosurgery, University of FloridaGainesville, FL, USA; ^5^Department of Veterans Affairs Rehabilitation Research and Development Center of Excellence at the Atlanta VA Medical CenterAtlanta, GA, USA; ^6^Department of Neurology, Emory UniversityAtlanta, GA, USA; ^7^Neurology Service/127, North Florida/South Georgia Veterans Health System and Department of Neurology, University of FloridaGainesville, FL, USA; ^8^McKnight Brain Institute, University of FloridaGainesville, FL, USA

**Keywords:** Broca's area, basal ganglia, thalamus, diffusion-weighted imaging, connectivity

## Abstract

In the recent decades structural connectivity between Broca's area and the basal ganglia has been postulated in the literature, though no direct evidence of this connectivity has yet been presented. The current study investigates this connectivity using a novel diffusion-weighted imaging (DWI) fiber tracking method in humans *in vivo*. Our findings suggest direct connections between sub-regions of Broca's area and the anterior one-third of the putamen, as well as the ventral anterior nucleus of the thalamus. Thus, we are the first to provide a detailed account of inferred circuitry involving basal ganglia, thalamus, and Broca's area, which would be a prerequisite to substantiate their support of language processing.

## Introduction

Language is a complex cognitive skill essential to many aspects of our everyday lives. Much effort has been dedicated to investigating the role and function of cortical substrates involved in language processing, most notably dominant hemisphere perisylvian cortex. Such work started over a century ago with luminaries such as Broca and Wernicke and has continued into the present (Broca, 1861; Wernicke, 1874). However, in recent decades evidence from lesion, neuropsychological, and neuroimaging studies suggests that the basal ganglia support language processing (Crosson, 1999; Copland et al., 2000a,b; Copland, 2003; Assaf et al., 2006). In particular, basal ganglia have been implicated to play a role in lexical selection and retrieval, various aspects of syntactic, morphological, and phonological processing, as well as higher-order language processing (Ullman, 2001, 2004; Friederici, 2002). Many of these functions also are commonly attributed to Broca's area (Amunts et al., 2004; Hagoort, 2005). Based on this relationship, a number of papers have postulated the existence of Broca's area basal ganglia thalamocortical circuitry (Brunner et al., 1982; Ullman, 2006). It is believed that this circuitry should closely resemble structural organization of other presently known prefrontal cortex basal ganglia loops (Alexander et al., 1986; Middleton and Strick, 2000a,b). Projections from Broca's area should enter the input nuclei of the basal ganglia circuitry, the caudate or the putamen, thence projecting to the globuspallidus and substantianigra. Connections between Broca's area and thalamic nuclei involved in language processing will comprise the cortico-thalamic and thalamocortical portion of the loop (Fisher, 1959; Schaltenbrand, 1965, 1975; Crosson et al., 2003). In particular, since Broca's area can be functionally segregated into two sub-regions—anterior involved in semantic processing and posterior involved with phonological and syntactical processing—two corticobasal ganglia loops may be present (Amunts et al., 2004). Although early studies delineating basal ganglia circuitry suggest that functionally distinct cortical regions should employ anatomically segregated loops, recent evidence demonstrates a great degree of convergence of cortical inputs within basal ganglia nuclei (Haber et al., 2006; Draganski et al., 2008). This convergence may play an important role in integrating information about different functional aspects of motor or cognitive domains in order to elicit the most contextually appropriate behavioral response (Draganski et al., 2008). In discourse, semantics and phonology are closely intertwined and may require integrated inputs from the two functional sub-regions of Broca's area to successfully select and execute contextually appropriate output. In particular, once an individual decides what he or she would like to say, this information must be integrated with the corresponding phonological representation of the statement in order to execute desired speech motor output.

Despite abundant evidence from human functional activity studies and limited anatomical evidence from non-human primates, no direct evidence of Broca's area-basal ganglia connectivity has yet been presented (Rizzolatti and Arbib, 1998; Rizzolatti et al., 2001; Ullman, 2001, 2004, 2006). To address this question, we used a new high-angular resolution diffusion-weighted imaging (HARDI) tractography method to trace connections between Broca's area and subcortical nuclei *in vivo* in human volunteers (Jian et al., 2007). Our tracking technique infers local fiber orientation by estimating probability associated with each direction based on the diffusion properties of the tissue. Multiple probability maxima are estimated for each voxel, thus allowing us to track crossing and branching fibers, which makes the current algorithm superior to traditional streamline tracking techniques based on the diffusion tensor models (Basser et al., 1994; Jian et al., 2007). Probabilistic tractography models are similar to our approach in that they model displacement probability distribution function for each potential fiber population (Behrens et al., 2003). These algorithms estimate probability distributions using Bayesian framework that requires complex solution techniques to determine the number of fiber populations in each voxel (Hosey et al., 2005; Behrens et al., 2007). Our approach models multiple fiber populations using a mixture of Wishart probability distributions where weights associated with each individual distribution are estimated directly from the data (Jian et al., 2007). These weights are used to recover the probability distribution function within each voxel and its maxima correspond to the preferred diffusion directions. We are the first, to our knowledge, to use this novel tracking approach in order to visualize the circuitry connecting Broca's area and the basal ganglia using an *in vivo* human model.

## Materials and methods

### Participants

Ten right-handed (two females), native English speakers, with no known neurological disorder were recruited. Age range of the participants was 19–35 years old (mean = 24.4, st.dev. = 4.62). Written informed consent was obtained from all participants in compliance with Institutional Review Board guidelines of the University of Florida and the North Florida/South Georgia Malcom Randall Veteran's Affairs Medical Center.

#### Image acquisition

All scans were obtained for each participant on a Philips Achieva 3T scanner (Amsterdam, Netherlands) using a 32-channel SENSE head coil. We acquired anatomical T1-weighted and DWI data on 10 healthy participants. Structural MP-RAGE T1-weighted scans were acquired with 130—1.0 mm sagittal slices, FOV = 240 mm (AP)—180 mm (FH), matrix = 256—192, TR = 9.90 ms, TE = 4.60 ms, Flip Angle = 8, voxel size = 1.0 mm × 0.94 mm × 0.94 mm. Diffusion-weighted images were acquired using single shot spin-echo echo planar imaging (EPI) with 60 × 2.0 mm axial slices (no gap), FOV = 224 mm (AP) × 224 mm (RL), matrix = 112 × 112, TR = 9509 ms, TE = 55 ms, Flip Angle = 90, voxel size = 2.0 × 2.0 × 2.0 mm, and time of acquisition = 5 min 42 s. The diffusion weighting gradients were isotropically distributed over a sphere using a 64-direction acquisition scheme with *b* = 1000 s/mm^2^. Six low *b*-value (*b* = 100 s/mm2) volumes were also collected. We acquire these low *b*-value images to ensure that we are not measuring signal attenuation from blood perfusion (Le Bihan et al., 1988). Low *b*-value scans also provide additional signal averages to improve the signal-to-noise resolution of our data. Two volumes with no diffusion weighting (*b* = 0) were also acquired with these parameters.

### Image processing

DWI data was corrected for eddy currents, skull stripped, and interpolated to 1 × 1 × 1 mm using FSL software package FMRIB software Library (www.fmrib.ox.ac.uk/fsl) (Jenkinson and Smith, 2001; Smith, 2002). We interpolate our data to 1 × 1 × 1 mm voxels as a means of increasing resolution for fiber tracking. Most conventional single direction streamline and probabilistic tractography methods either interpolate the vector field or streamline propagation vectors. In the first case, interpolation ensures that the tensor vector field is continuous and smooth (Mori et al., 1999). In the second case, interpolation of the streamline vectors provides a consistent method for choosing propagation direction (Behrens et al., 2003, 2007). Our tracking method traces pathways through a multi-directional field, which makes interpolation of the propagation vectors difficult. To overcome this obstacle, we interpolate the original data as part of pre-processing as described below.

### Tractography method

#### Fiber orientation estimation

For each brain voxel the displacement probability function (PDF) is estimated using the Method of Wishart (Jian and Vemuri, 2007a,b; Jian et al., 2007), implemented using an in-house software package written in IDL (Exelis Visual Information Systems, Boulder, CO). In each voxel, a PDF is defined on the space of 3 × 3 symmetric positive definite matrices (e.g., rank-2 diffusion tensors). Employing a Wishart distribution to represent this displacement probability as a distribution of matrices leads to a Laplace transform formulation of the diffusion weighted signal. This formulation provides a convenient closed-form expression which allows the inverse problem to be posed in a deconvolution framework.

To solve the deconvolution problem, we proceed as outlined by Jian and colleagues (Jian and Vemuri, 2007a). First we create 162 diffusion tensors, each with a principle eigenvector evenly spaced on the unit sphere with eigenvalues (1.5, 0.4, 0.4) μ^2^/ms. The measured signal is represented by a weighted sum of terms which depend on these tensors. Estimation of the vector of weights is performed by solving the linear system (Equation 6; Jian and Vemuri, 2007a) using non-negative least squares (NNLS). In a comparison of optimization methods, NNLS was found to perform well in the presence of noise and it yields a sparse solution (Jian and Vemuri, 2007a). Once the weights are recovered, the displacement probability distribution, PDF, is estimated, using Equation 14 (Jian and Vemuri, 2007a), at 642 points on the unit sphere, with *t* = Δ − δ/3, and *r* = 12 μm.

#### Determination of maxima

Once the diffusion displacement PDF has been estimated, the peaks of this probability profile are extracted using a gradient ascent optimization scheme with multiple restarts. The initial choice of 42 input starting points, based on the tessellation of an icosahedron over the spherical domain, is used as input to the optimization. The following procedure is applied for each input point: (1) the probability distribution is evaluated at the input point and at each of its neighboring PDF points, (2) Comparing these values, the starting point is then moved in the direction of greatest increase (i.e., gradient ascent) in probability, (3) then this process is iterated until a local maximum is reached. Once all of the input points are processed, the maxima are corrected for antipodal symmetry and duplicate maxima are deleted. The surviving maxima are sorted by probability value and recorded for use as tracking directions.

#### Seeding and tracking

The entire brain is densely seeded with 64 equidistant points in each voxel. The seed points are distributed so as to create a uniform field of seed points throughout the brain that is consistent across voxel boundaries. From each seed point, a streamline is launched bi-directionally along paths defined by a maxima in the PDF. The streamline front is stepped using Euler integration in increments of 0.25 mm by choosing, at each voxel, the estimated fiber direction from maxima in the PDF that is most inline with the streamline's current direction of travel. In the case of a tie, the direction with the highest PDF value is used. Streamlines progress in this way until either they are required to make a turn with an angle greater than 50°, or if they reach the boundary of the brain mask. Fractional anisotropy (FA) is not used as a stopping criterion. FA is a scalar that has values between 0 and 1 and represents local diffusion properties of the tissue. An FA value of 0 means that diffusion is isotropic and does not follow any given preferred direction. FA values close to 1 indicate that diffusion is anisotropic which means that there exists a single preferred diffusion orientation. FA is ambiguous in complex tissue regions with fiber heterogeneity (i.e., “kissing” or “crossing” fibers) and does not accurately represent the tissue. Since the pathways that we are interested in must pass through many regions of complex white matter architecture we did not use FA values as a stopping criterion. The resulting streamlines are filtered for connectivity between brain regions by checking if a point on the streamline touches both regions of interest.

#### Tracking visual pathways

In order to ensure that the tracking method allows accurate resolution of crossing and branching fibers, we tested our technique by tracing visual pathways between the optic chiasm, the lateral geniculate nucleus (LGN) of the thalamus, and the occipital cortex. In order to accurately identify the LGN on our structural images, we created a mask of the optic chiasm and traced pathways passing through this region of interest. To accomplish this, we applied the chiasm mask to the whole brain tractography results to filter out pathways traversing the chiasm. Next, we created a mask of the LGN by identifying the termination the optic tract within the left hemisphere of participant 3. In order to trace the optic radiations, we created a mask of the occipital cortex using a single coronal slice through this region located 10 mm anterior of the posterior border of the occipital lobe on the skull stripped T1-weighted image of participant 3. Finally we merged the above-mentioned masks into a single file and used this combined mask to filter out pathways that pass through all three regions. Resulting visual pathways are depicted in Figure 6. Top panel shows the axial view of the optic tract and the optic radiations with the LGN shown in blue. The bottom panel shows a 3-dimentional anterior-superior view of the pathways. We note that our method successfully allows us to resolve both superior and inferior portions of the optic radiations, which indicates that our data and our tracking technique provide necessary resolution to resolve crossing and branching fiber pathways.

Moreover, these results suggest that if projections between pars triangularis/opercularis terminate in distinct locations within putamen, our tracking method should be able to resolve these termination points. Our findings show that connections from pars triangularis and pars opercularis share common termination locations within the anterior one-third of the putamen in all ten of our participants. Although we cannot definitively state that pars triangularis/opercularis connect with the same regions of putamen, our results show that their termination are overlapping. Further studies employing alternative methods (i.e., tracer injections) are necessary to better quantify the amount of tissue within putamen that shares direct connectivity with pars triangularis and pars opercularis.

In addition, we wanted to test the likelihood of obtaining a false positive result using our tractography method (i.e., whether our tracking method will generate pathways between regions that do not share direct anatomical connectivity). To do so we attempted to trace pathways between globuspallidus and pars triangularis/opercularis in five randomly selected participants from our study (participants #2, 3, 6, 7, and 8). We created a mask of globuspallidus using FSL FIST module (Patenaude et al., 2011). This mask was registered to the diffusion space of each of the five participants applying the same linear transform used to register the other subcortical masks (caudate, putamen, and thalamus). The resulting globuspallidus mask was applied to the whole brain tractography results to infer all of the pathways passing through this region. Resulting pathways consisted of connections between globuspallidus and other subcortical structures (i.e., striatum, thalamus) and their cortical projection sites. Next, we intersected these pathways with pars triangularis and pars opercularis masks to infer pathways connecting these cortical areas with globuspallidus. In addition, we used putamen and thalamus masks as exclusion masks to filter out pathways coursing through these subcortical structures and preserve only the pathways directly connecting Broca's area and globuspallidus. As a result of this analysis, we found that our tracking method does not generate pathways directly connecting cortical regions of interest with globuspallidus, a finding supported by animal tracer studies (Alexander et al., 1986; Middleton and Strick, 2000a). Although formal null hypothesis testing is not presently available for tractography studies, our results show that our tracking method is able to accurately infer known anatomical patterns of connectivity.

### Regions of interest

Cortical masks of pars triangularis and pars opercularis were drawn on the T1-weighted images and registered to diffusion space using the FSL FLIRT module (Jenkinson and Smith, 2001). The lateral-most sagittal slice of the frontal cortex of the skull-stripped T1-weighted scan was used as the lateral border of the cortical masks. The medial border was defined by the first sagittal slice traversing the insular cortex. The dorsal border of the masks was defined by the inferior frontal sulcus, while by the ventral border was defined by the Sylvian fissure. The anterior border of the pars triangularis mask was defined by a coronal plane through the anterior margin of the anterior horizontal ramus of the Sylvian fissure, and its posterior border was defined by the anterior ascending ramus of the Sylvian fissure. The anterior border of the pars opercularis mask was drawn by following the posterior border of the pars triangularis mask, leaving one voxel distance between the two masks to ensure that they are non-overlapping. The posterior border of pars opercularis mask was defined by the inferior precentral sulcus.

**Figure 1:**
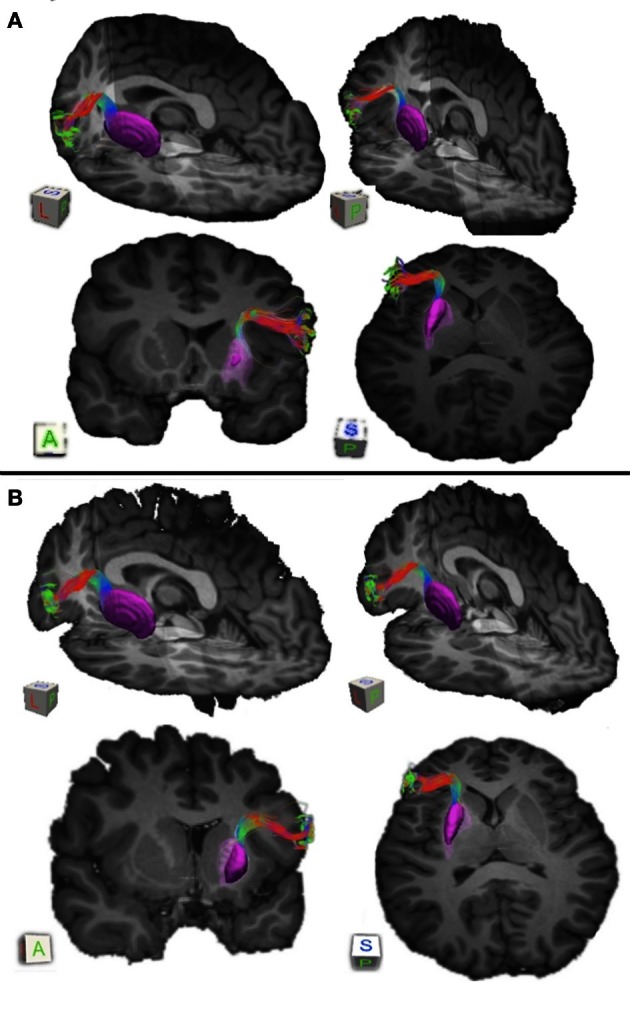
**Pathways connecting pars triangularis (A) and pars opercularis (B) and anterior putamen (purple) in a representative participant (participant 3).** Color gradient of pathways represents directionality of fiber orientation: red left-right, green anterior-posterior, blue superior-inferior. The cubes located to the right each image represent spatial orientation of the brain. Letters on the faces of the cubes represent directions: A, anterior; P, posterior; S, superior; L, left. The images depict a single participant (participant 3).

**Figure 2:**
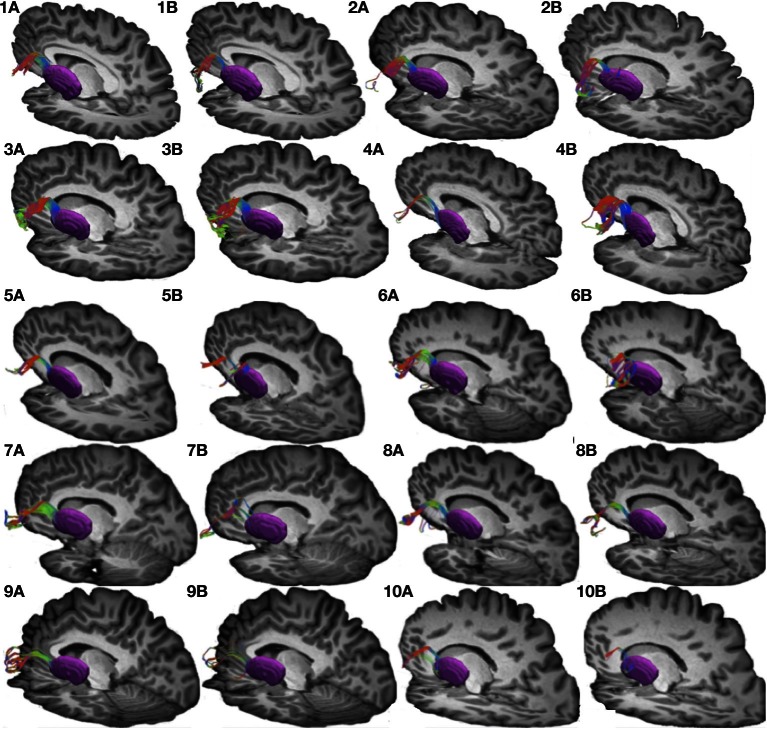
**Connections between pars triangularis (A) and pars opercularis (B) and putamen (purple) in each of our ten participants.** Color gradient represents the same directionality as in Figure 1.

**Figure 3:**
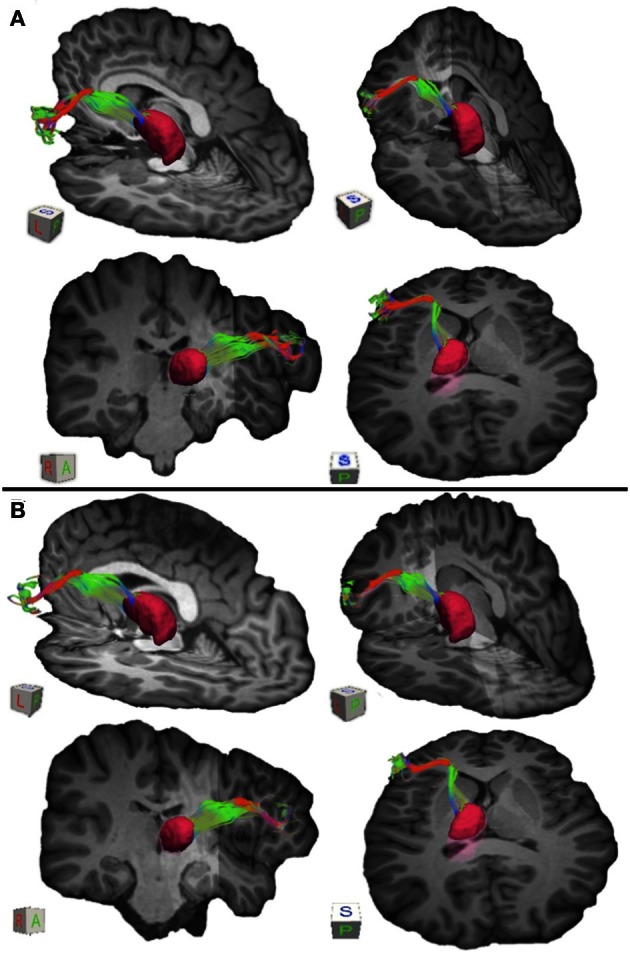
**Connections between pars triangularis (A) and pars opercularis (B) and the thalamus (pink) in a representative participant (participant 3).** Color gradient of the pathways and letters on the faces of the cubes have the same designation as in Figure 1.

**Figure 4:**
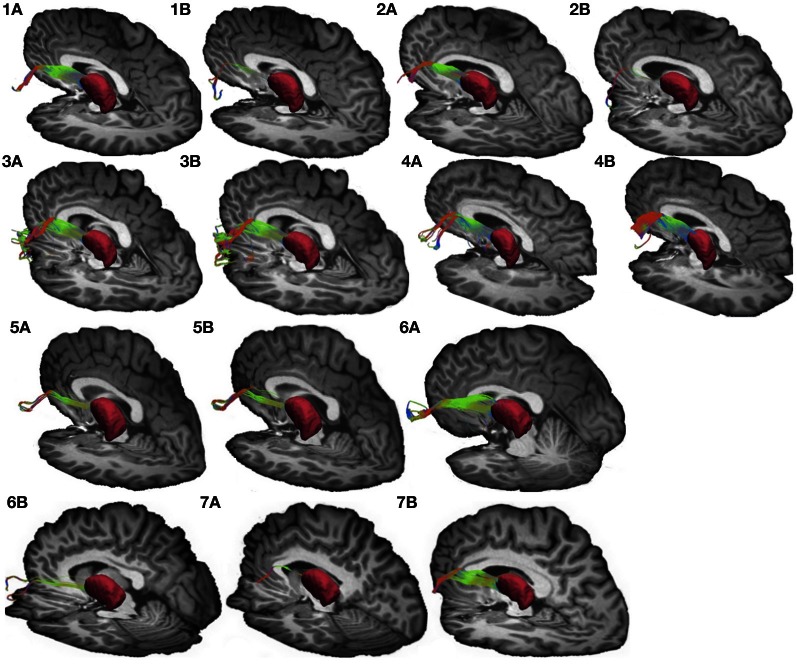
**Connections between pars triangularis (A) and pars opercularis (B) and the thalamus (pink) in our ten participants.** Color gradient represents the same directionality as in Figure 1.

We used the FSL FIRST module to perform subcortical segmentation and identify caudate, putamen, and thalamus for each participant using their T1-weighted images (Patenaude et al., 2011). Subcortical masks were then registered to the native diffusion space of each participant using FLIRT (Jenkinson and Smith, 2001). Putamen masks for each participant can be seen in Figures 1 and 2 (purple mask), while thalamic masks can be seen in Figures 3 and 4 (pink mask). We used our cortical and subcortical masks to render tracts connecting these regions. To do so, we first applied each cortical mask to the whole brain tractography results to filter tracts originating or terminating in a given cortical region. Next, we intersected the resulting pathways with each subcortical mask to infer tracts connecting pars triangularis and pars opercularis with the corresponding subcortical structure. Tracing of these pathways terminated upon reaching each of the subcortical masks.

## Results

To investigate connectivity between Broca's area and the basal ganglia we created two cortical regions of interest—pars opercularis and pars triangularis—as well as three subcortical masks consisting of the caudate, putamen and thalamus (see Materials and Methods for more details). We used our tracking algorithm to trace white matter fibers between these cortical and subcortical regions. The inherent spatial resolution of diffusion data limits the size of a fiber bundle that can be traced using tractography. Thus, we focused our investigation of the Broca's area basal ganglia circuitry on relatively large tracts: the input portion of a basal ganglia loop connecting Broca's area with either caudate or putamen and the output portion connecting Broca's area with the thalamus.

### Connectivity between broca's area and the input nuclei of the basal ganglia

Our results show strong direct connectivity between Broca's area and the anterior one-third of the putamen that was found consistently across all ten of our participants. Figure 1 depicts a representative rendering of the fiber bundles connecting pars triangularis (A) and pars opercularis (B) with putamen (purple) in a single participant (participant 3), while Figure 2 depicts these pathways in all ten participants. The color gradient represents local fiber orientation: red left-right, green anterior-posterior, and blue superior-inferior. Projections from both pars triangularis and pars opercularis course medially, passing over and around an anterior, superior portion of circular sulcus, and after passing the insula, make a nearly ninety-degree turn to then travel posteriorly, and descend down into putamen. Projections from pars triangularis and pars opercularis enter the anterior-superior one third of the putamen in all participants. The relative volumes of the two fiber bundles are similar, with the exception of participants 4, 7, and 9 (participant 4 demonstrated more prominent pars opercularis-putamen projections, and the reverse was true for participants 7 and 9). Table 1 below shows that participants 4 and 10 had the smallest tract volume for pathways connecting pars triangularis and putamen (766 mm^3^ and 562 mm^3^ respectively). The average volume for this inferred pathway is 1517.5 mm^3^ (st.dev = 661.4 mm^3^). For pathways connecting pars opercularis and putamen, participants 7 and 10 has the smallest track volume (605 mm^3^ and 379 mm^3^ respectively). The average track volume for this pathway was 1340.6 mm^3^ (st.dev = 711.3 mm^3^).

**Table 1:** **Inferred track volumes for pathways connecting Broca's area, putamen, and thalamus**.

**Participant**	**Pars triangularis—putamen track volume (mm^3^)**	**Pars triangularis—thalamus track volume (mm^3^)**	**Pars opercularis—putamen track volume (mm^3^)**	**Pars opercularis—thalamus track volume (mm^3^)**
1	1913	2594	1337	342
2	1539	1730	1148	382
3	2439	3439	2468	2996
4	766	2479	2083	2045
5	841	863	811	901
6	2482	0	1534	0
7	1341	1849	605	0
8	1622	0	870	0
9	1670	1302	2171	170
10	562	1874	379	0

Within the anterior one-third of the putamen, projection sites from pars opercularis and pars triangularis do not appear to be segregated, as both fiber pathways entered the nucleus within a similar overlapping location. In order to ensure that this convergence is not due to either a lack of resolution of our data or difficulty tracking pathways through gray matter using our tracking approach, we created a mask of the lateral geniculate nucleus of the thalamus, a small gray matter nucleus located deep within the brain, and traced fiber bundles coursing through this region (see Figure 6). As a result, we were able to successfully resolve connection sites of the optic tract, inferior portion of the optic radiations (the Meyers loop), as well as the superior portion of the optic radiations (Baum's loop). The ability to resolve complex fiber bundle organization within a gray matter region much smaller than the basal ganglia nuclei examined in the present study suggests that convergence of inputs from pars triangularis and pars opercularis within anterior putamen revealed here is a genuine characteristic of this circuitry.

Tracking between either pars triangularis or pars opercularis and the caudate did not reveal any pathways connecting these regions. Our analysis demonstrated that some of the pathways connecting pars triangularis/opercularis and thalamus travel close to or in some cases approach the surface of the caudate nucleus. However, application of the thalamus mask as an exclusion mask eliminates all of these pathways, indicating that there are no tracts that travel between Broca's area and caudate exclusively.

### Connectivity between Broca's area and the thalamus

To visualize the output portion of the Broca's area basal ganglia thalamocortical circuitry, we tracked projections between pars triangularis and pars opercularis and the thalamus. Figure 3 represents three-dimensional renderings of the fiber pathways connecting the cortical regions with the thalamus in a single participant (participant 3) (thalamus depicted in pink color; color gradient of the fibers represents fiber orientation as in Figure 1); Figure 4 represents these pathways in all participants. Pars triangularis (A) and pars opercularis (B) tracts course medially, and then take an obtuse angle to travel posteriorly toward the thalamus. The projection site of the two fiber bundles is located within the ventral anterior nucleus of the thalamus for all participants in whom these projections could be traced. We note that we were unable to trace connections between Broca's area and the thalamus in participants 6 and 8. In addition, we could not trace projections between pars opercularis and the thalamus in participants 7 and 10. Table 1 represents track volumes in cubic millimeters for the inferred pathways in our ten participants. The average track volume for the inferred pathways connecting pars triangularis and thalamus is 1613 mm^3^ (st.dev = 1107.9 mm^3^) and 778.2 mm^3^ (st.dev = 1032.2 mm^3^) for pars opercularis thalamic pathways.

Overall, we note that pathways connecting Broca's area with the thalamus were more variable than pathways connecting Broca's area and putamen in our dataset. We believe that this variability may be present because the angle that the pathways traverse exceeds that allowed by our tracking algorithm to ensure that the tracts do not loop back onto themselves (see section Seeding and Tracking in Materials and Methods). Applying larger turning angle with the current data resolution would result in tracts that return to the voxels from which they originated. Future studies employing higher resolution data acquisition schemes and tracking methods allowing greater deviation in step angle may be necessary to reduce variability observed in the present study.

## Discussion

**Figure 5:**
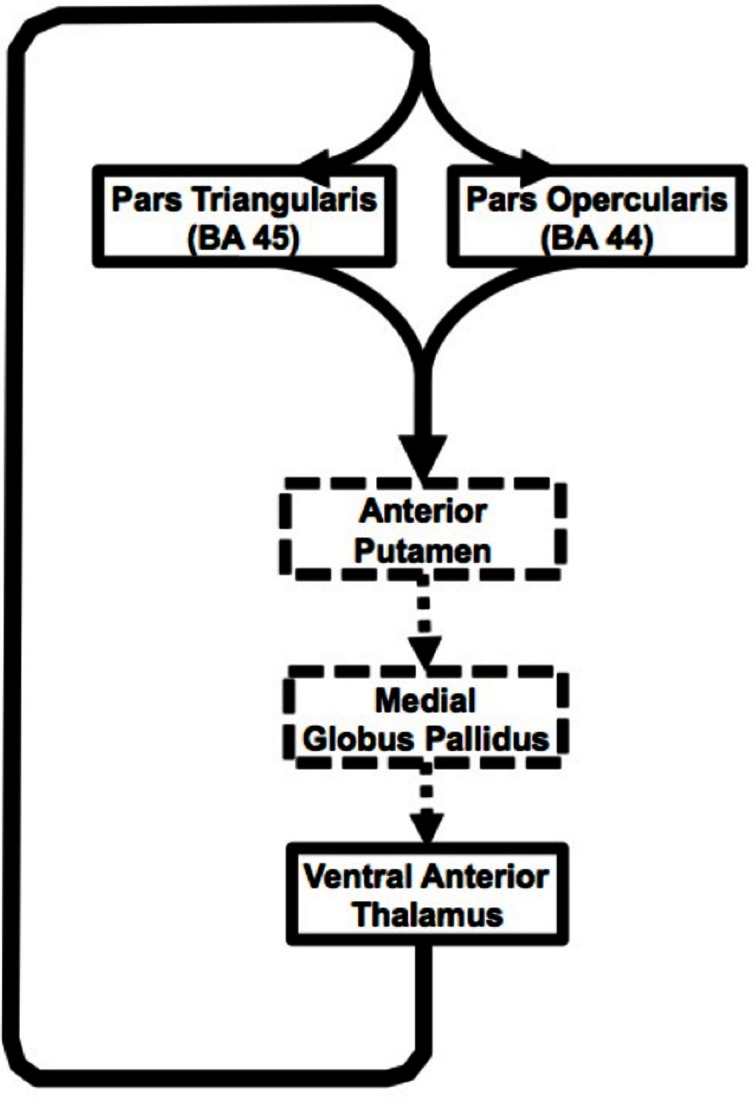
**Schematic representation of the Broca's area basal ganglia loops.** Solid black arrow represent pathways presented in our study, dashed arrows represent inferred based on other studies.

The present study provides a detailed description of the structure and organization of Broca's area-basal ganglia circuitry in humans *in vivo* (see Figure 5 for a schematic rendering of this circuitry). In particular, the study addressed corticostriatal and thalamocortical portions of the circuitry. Our findings present a major contribution to the study of basal ganglia thalamic connectivity within the human brain, as we are the first, to our knowledge, to provide evidence of direct connectivity between Broca's area and the basal ganglia. In recent decades, it became apparent that although basal ganglia are not involved in primary language functions, these structures play a crucial role in increasing the signal-to-noise ratio during actions (Mink, 1996; Nambu et al., 2000), which can be applied to language processing. It has been suggested that the basal ganglia accomplish this function by enhancing cortical signal for selected items and suppressing cortical signal for competing items (Crosson et al., 2007). Our investigation of this connectivity demonstrates that Broca's area employs anterior putamen as its input nucleus to the basal ganglia. Although the putamen often is considered a motor nucleus, both the dorsal and ventral lateral prefrontal cortex of macaques project to the anterior (precommissural) putamen (Selemon and Goldman-Rakic, 1985). For humans, this region has been implicated in a number of language processing domains, in particular, phonological processing (Devlin et al., 2003; Tettamanti et al., 2005), reading (Seghier and Price, 2010), semantic processing (Devlin et al., 2003), and semantic priming (Rossell et al., 2001). Functional connectivity studies have demonstrated direct functional connectivity between Broca's area and putamen and suggested that this circuitry is involved in articulatory control and initiation of phonological representations (Booth et al., 2007). Additionally, direct electrode stimulation of the anterior putamen results in temporary speech deficits (Robles et al., 2005), while activation of this region during word repetition (Wise et al., 1999; Wildgruber et al., 2001) suggests its involvement in speech articulation. Broca's area, in particular its posterior aspect corresponding to pars opercularis, has been shown to be involved in phonological and syntactical processing, and speech production, as well as non-linguistic laryngeal control (Friederici, 2002; Devlin et al., 2003; Amunts et al., 2004; Hagoort, 2005). Therefore, one of the functions that direct connectivity of these regions may support is sharpening the activation for the most contextually appropriate phonemes and their corresponding motor programming during discourse. Portions of the prefrontal cortex including Broca's area together with basal ganglia are thought to be a part of a procedural system that subserves “computational ‘mental grammar’,” involved in the combination of relevant phonemes into meaningful structures, such as words (Ullman, 2001; Booth et al., 2007). Thus, once the most semantically pertinent response has been selected, functional networks involving posterior Broca's area basal ganglia circuitry may be engaged to activate appropriate phonemes and their articulatory programs to produce the selected response.

The anterior portion of Broca's area corresponding to pars triangularis also showed direct connectivity with anterior putamen in our study. This portion of Broca's area is more involved in semantic than phonological processing (Devlin et al., 2003). The region within the anterior putamen that serves as a projection site of the tracts originating within pars triangularis greatly overlaps with that of the tracts originating in pars opercularis. Although classical models of basal ganglia connectivity with the cortex suggest that the projection sites within the basal ganglia originating from functionally distinct cortical regions should be segregated, recent evidence demonstrates a high degree of convergence of these projections within the striatum (Haber et al., 2006; Draganski et al., 2008). This evidence arises from studies using tracer injections in non-human primates and non-invasive tractography studies performed *in vivo* in humans. In particular, Haber and colleagues demonstrated the convergence within the striatum of projections from cingulate and orbitofrontal cortical region of non-human primates involved in reward processing and tracts originating in the dorsolateral prefrontal cortex implicated in many aspects of cognitive processing converge within the striatum (Haber et al., 2006). This finding was later replicated in the human brain using a tractography method similar to one used in the present study (Draganski et al., 2008). These results suggest that the striatum may be mediating reward-based learning by integrating reward representation from orbito-striatal network with optimal contextual behavioral output within the dorsolateral prefrontal-striatal circuitry.

In the present study, two functionally distinct regions within Broca's area, the anterior portion implicated in semantic processing and posterior portion involved in phonology and syntax, project to an overlapping region within the anterior putamen. Convergence of these inputs may serve particular functional significance for language processing. We hypothesize that once the most appropriate semantic response has been enhanced through interactions within the pars triangularis basal ganglia circuitry, this selection is relayed to the pars opercularis network to strengthen activation for the corresponding lexical-phonological representation. This ensures that the desired semantic response is articulated using appropriate phonemes during discourse. The overlap of these circuits at the level of the anterior putamen and downstream portions of the loop, including the ventral anterior thalamus, may ensure that corresponding semantic and lexical-phonological representations are fine-tuned during word selection.

It also is worth noting that cortico-thalamo-cortical circuitry has been demonstrated (Llano et al., 2009) and that one area of cortex may activate another closely related area of cortex through the thalamus, independent of their cortico-cortical connections (Theyel et al., 2010). Cortico-thalamic connections originating from cortical layer 6 are thought to be involved in changing thalamic neurons between high- and low-fidelity transfer modes of function, while cortico-thalamic connections originating from cortical layer 5 are thought to be involved in information transfer between cortical areas (Sherman and Guillery, 2006; Theyel et al., 2010). Crosson (2012) has discussed the implications of these different mechanisms for language. Hence, pars triangularis and pars opercularis also may communicate through cortico-thalamo-cortical pathways represented by our current findings, as well as through basal ganglia loops.

In summary, current findings suggest input from two adjacent cortical areas (pars triangularis and pars opercularis) subserving different, but closely related language functions converge on the same region of the anterior putamen. Similarly, our findings suggest that output from the thalamic portion of the loop comes from a single thalamic region, but innervates both of these closely related cortical areas (Figure 5). This arrangement would serve to simultaneously strengthen corresponding lexical and semantic representations during word selection. The portions of the loops that the current study did not address are represented with dashed lines. Cortical-thalamo-cortical mechanisms may also be represented in the pathways between pars opercularis or pars triangularis and the thalamus.

### Technical limitations

**Figure 6:**
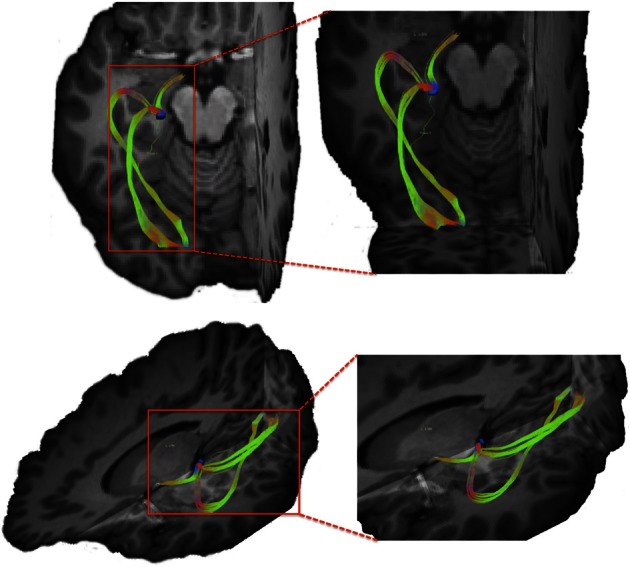
**Visual pathways resulting from tracking between lateral geniculate nucleus (LGN) and the occipital cortex.** We note that using our tracking technique we are able to resolve both superior and inferior branches of the optic radiations originating within the LGN.

The present study of Broca's area-basal ganglia circuitry is not, however, without limitations. Inherent resolution of the currently available diffusion-weighted data did not allow us to track striato-pallido-thalamic projections of the basal ganglia loops in question. Higher resolution may allow better visualization of these fiber tracts, but increasing the resolution adds more noise and therefore more uncertainty to the data, increasing tracking difficulty. We believe, however, that the current data resolution (2 mm isotropic voxels) provides sufficient detail to infer pathways connecting Broca's area and subcortical structures using our tractography approach. Each pathway connecting Broca's area and subcortical structures investigated in the current study extended more than 2 mm in both superior-inferior and medial-lateral directions. The pathways took the most direct route between the cortex and subcortical structures, and their trajectories followed known anatomical landmarks. Furthermore, our tractography analysis of pathways coursing through the LGN provide additional support that our diffusion data was of sufficient resolution to allow inference of complex white matter architecture within subcortical regions of interest (Figure 6).

Another limitation is the fact that the currently available methodological approaches to analyzing diffusion-weighted data do not allow us to infer fiber polarity (e.g., whether a pathway in question is cortico thalamic or thalamocortical). Functional connectivity approaches could use methods such as dynamic causal modeling to infer whether activity of one region drives activity changes in other regions within the same network and may be helpful in determining directionality of these connections. This approach, used in conjunction with the tracking method employed in the present study, could be used to resolve the extent to which convergence of the two Broca's area basal ganglia loops mediates cortical activity changes within pars triangularis and pars opercularis. If our conclusions based on anatomical evidence presented here are correct, the anterior putamen and ventral anterior thalamus should demonstrate functional connectivity with Broca's area during tasks involving semantic and phonological processing. Partial support for this claim is provided by the evidence that anterior putamen does modulate activation within inferior frontal gyrus during reading (Booth et al., 2007). However, additional tasks engaging semantic processing, such as category member generation, are crucial to parcel out the interface of semantics and phonology within the Broca's area basal ganglia circuitry. Lemaire and colleagues devised such a study by combining functional activation clusters from an antonym-generation fMRI task with diffusion tractography (Lemaire et al., 2012). The authors demonstrated complex structural connectivity between Broca's area and other cortical zones involved in language processing supported by previous data from our laboratory (Ford et al., 2010). The innovative approach devised by Lemaire and colleagues should be implemented in future studies to infer functional significance of structural connectivity between Broca's area and subcortical structures presented in this study.

Another limitation inherent to this and other diffusion tractography studies is lack of explicitly defined null hypothesis (Jbabdi and Johansen-Berg, 2011). Ideally, we would like to be able to perform formal statistical testing on results generated by a tracking algorithm controlling for type I and type II errors. However, to accomplish this task we must first establish an appropriate null distribution within each data voxel that would support the null hypothesis (i.e., no tracts between region A and region B exist). Presently, no such distribution has been formulated, although efforts are now being made to accomplish this task (Morris et al., 2008). In an effort to overcome this limitation in the present study, we performed an additional analysis examining whether our tracking method would generate a false positive result by attempting to trace pathways that are not believed to be present based on the animal tracer literature (see section Tracking Visual Pathways in Materials and Methods for more detail). We attempted to trace pathways between globuspallidus and pars triangularis/opercularis. Globus pallidus is not believed to share direct connectivity with the cortex, but rather these connections are mediated by the striatum on the input side and by the thalamus on the output side (Alexander et al., 1986; Middleton and Strick, 2000a). Our tracking algorithm did not generate any pathways directly connecting globuspallidus and pars triangularis/opercularis, providing further support for sensitivity of our method. It is important to note that globuspallidus is typically difficult to resolve in conventional functional MR imaging due susceptibility artifacts imposed by iron deposits within this region. Our data, however, did not show significant signal drop out within this structure. Moreover, our tracking results showed pathways connecting globuspallidus with striatum and thalamus indicating that our data had sufficient signal-to-noise ratio characteristics to carry out tractography analysis.

Our results showed a considerable amount of individual variability in track volumes for pathways connecting Broca's area with thalamic nuclei. We believe that this variability may stem from a number of different factors. First, this variability may be due to individual differences in white matter organization. This hypothesis is supported by findings that individual patterns of gyral and sulcal distributions, as well as the underlying cytorachitectonic divisions, vary a lot between different individuals (Amunts et al., 1999). Given that our tractography approach was guided by cortical and subcortical grey matter regions of interest, variability in size and location of these regions introduces variability in trajectories and size of the resulting white matter pathways.

Another potential explanation for variability in tract volumes reported in this study is susceptibility of diffusion-weighted data to motion-related artifacts. The inherent resolution of our diffusion-weighted data imposes considerable constraints on the amount of motion that could be present in a dataset without affecting tractography results. Our acquisition resolution in this study was 2 × 2 × 2 mm^3^, which suggests that any movement larger than 2 mm would result signal averaging between neighboring voxels. Spatial signal averaging is further enhanced by motion correction algorithms that attempt to correct spatial misalignments by registering diffusion-weighted volumes to each other. If a participant moves by 2 mm or more during acquisition of one or more diffusion-weighted data volumes the final motion-corrected dataset could contain substantial amount of spatial averaging from the affected volumes. Unfortunately, since all of our participants were scanned without sedation and with minimal constriction of head and neck to maintain participants' comfort, motion in the range of 2–5 mm is not unusual. Data acquisition of excised tissue would eliminate motion related artifacts, and future studiesare necessary to further investigate these effects. Miller and colleagues provide an excellent example of an ex vivo data acquisition and analysis using diffusion-weighted tractography (Miller et al., 2011). The authors infer trajectories and structural characteristics of well-understood white matter pathways (corticospinal tracts, corpus callosum, cingulum, and fornix) using sub-millimeter diffusion data. This high resolution acquisition approach could be extended to pathways examined in the present study to further infer structural connectivity of Broca's area and subcortical structures. Studies employing ex vivo diffusion acquisitions should be designed with caution however. As Miller and colleagues point out, diffusion indices measured *ex vivo* depart significantly from *in vivo* measurements. In particular, fractional anisotropy and diffusivity values are significantly lower that those commonly found *in vivo* data, and they correlate significantly with time period between death and fixation (Miller et al., 2011). Significantly lower diffusion indices may result in poorer grey/white matter differentiation, and increased uncertainty in principal diffusion orientation may present addition complexities for *ex vivo* tractography. Type and concentration of fixative used, fixative washout method and time period prior to MR acquisition, and tissue pathology are some additional factors that future investigators must be aware of prior to conducting ex vivo diffusion data acquisitions. As stated by Miller and colleagues, control human post mortem samples are difficult to obtain as vast majority of fixed brains are preserved to investigate brain pathologies (Miller et al., 2011). Alternative image acquisition techniques could be employed to complement *ex vivo* diffusion data and address some of difficulties associated with post mortem diffusion acquisitions. Specifically, Axer and colleagues used polarized light imaging (PLI) to quantify fiber orientation in histological brain sections (Axer et al., 2011). This method offers promising future directions in comparing diffusion MR data findings with histology.

Tractography has provided many advances in our understanding of neural connectivity; however, given its limitations, we must interpret results of tractography studies with caution. Pathways delineated by tracking algorithms do not directly represent actual neural fibers, but rather are our best estimate of fiber trajectories inferred based on local diffusion properties of the tissues. Tractography users should heavily scrutinize resulting pathways and determine their validity based on prior anatomical knowledge (Jbabdi and Johansen-Berg, 2011). In addition, efforts should be made to correlate statistical measures associated with tractography (i.e., tract volume measures) with behavioral/cognitive observations to further relate anatomical findings with functional results.

Despite its limitations diffusion tractography remains the only presently available technique to examine structural connectivity *in vivo*. Post-mortem injection of fluorescent dyes allows tracing of the fibers for only tens of millimeters, largely restricting the types of pathways that could be examined using this method (Mufson et al., 1990). Dissection studies are better suited for delineation of long-distance pathways; however, successful demarcation of small crossing and/or collinear tracts is often difficult and frequently requires additional histological information (Van Buren and Burke, 1972). Animal tracer studies have provided a large body of knowledge about neural circuitry and are regarded as the gold standard to study structural connectivity (Alexander et al., 1986; Middleton and Strick, 2000a,b; Llano et al., 2009). However, to our knowledge, connections between macaque BA 44/45 and the basal ganglia have not yet been investigated using this method. Moreover, advanced language faculty is unique to humans and connectivity patterns derived from animal literature may not accurately reflect human language networks. In fact, comparative studies demonstrated prominent differences in connectivity patterns of ventral prefrontal cortex between human, chimpanzee, and macaque brains (Rilling et al., 2008; Thiebaut De Schotten et al., 2012).

In conclusion, the present study describes the probable structure and organization of Broca's area basal ganglia loops in human brain. These results suggest that pars triangularis and pars opercularis project to the anterior putamen and also connect with the ventral anterior nucleus of the thalamus. We propose that the network involving these cortical and subcortical regions could be involved in sharpening activation of the contextually appropriate semantic response and its corresponding lexical-phonological representation. Pars triangularis-basal ganglia pathways may enhance activation of the most semantically pertinent response, while suppressing other category members. Selection of the most semantically fitting response is integrated with its corresponding phonemic representation through converging inputs from pars triangularis and pars opercularis within anterior putamen and ventral anterior thalamus.

## Author contributions

Anastasia A. Ford, Bruce Crosson and Keith White conceptualized the experiment, Anastasia A. Ford carried out all data analysis and wrote the paper. William Triplett and Thomas Mareci developed the software used in the experiment. Atchar Sudhyadhom, Keith McGregor, Joseph Gullett, David B. FitzGerald, and Anastasia A. Ford collected the data.

### Conflict of interest statement

The authors declare that the research was conducted in the absence of any commercial or financial relationships that could be construed as a potential conflict of interest.
